# A Case Report of a Large Splenic Cyst in a Pediatric Patient

**DOI:** 10.7759/cureus.46113

**Published:** 2023-09-28

**Authors:** Rawan Alharbi, Rudayna Almohammdi, Weam Alharbi, Farah Alshaikhjafar, Ahlam Alharbi

**Affiliations:** 1 Medicine, Jordan University of Science and Technology, Irbid, JOR; 2 Medicine, Al-Rayan Colleges, Madinah, SAU; 3 Medicine, Taibah University, Madinah, SAU; 4 Medicine, Al-Baha University, Al-Baha, SAU; 5 Family Medicine, Primary Health Care Center, Riyadh, SAU

**Keywords:** case report, computed tomography, abdominal pain, epithelial cyst, spleen

## Abstract

Splenic cysts in the pediatric population are rare but can present with a range of clinical manifestations. Acute abdominal pain, although uncommon, is a significant presentation that requires prompt evaluation and management. We present the case of an 11-year-old female who presented to the emergency department with severe left upper quadrant abdominal pain of 24 hours’ duration. Physical examination revealed tenderness and guarding in the left upper quadrant with a palpable, firm mass. Elevated serum amylase and lipase levels initially raised suspicion of a pancreatic etiology, but further investigations confirmed the presence of a large cystic lesion in the spleen. The patient underwent laparoscopic splenectomy, and the resected specimen confirmed a benign splenic cyst. Postoperatively, she recovered uneventfully and was discharged with appropriate follow-up plans. This case report underscores the importance of early recognition and prompt surgical intervention in managing splenic cysts in pediatric patients. The diverse etiologies and pathophysiological mechanisms of splenic cysts necessitate a comprehensive diagnostic approach.

## Introduction

Splenic cysts in the pediatric population are relatively rare but can present with a wide range of clinical manifestations [1]. Among these, acute abdominal pain is an uncommon but important presentation, necessitating prompt evaluation and management. This case report highlights the clinical encounter of an 11-year-old girl who presented with acute abdominal pain.

Splenic cysts can be categorized into various types, including true cysts, pseudocysts, parasitic cysts, and neoplastic cysts [1]. The etiology of these cysts varies and may involve congenital, traumatic, inflammatory, or neoplastic factors. Accurate diagnosis is crucial, as treatment strategies depend on the type, size, and symptomatic presentation of the cyst [2].

## Case presentation

An 11-year-old female patient was brought to the emergency department with a complaint of sudden and severe abdominal pain of 24 hours’ duration. The patient had no significant past medical history and was previously in good health. Her parents reported that the pain had progressively worsened and was located in the left upper quadrant of the abdomen. There was no history of trauma or recent illness. The patient denied any fever, vomiting, diarrhea, or changes in bowel habits.

Upon physical examination, the patient appeared uncomfortable and in distress. She was afebrile with a heart rate of 98 beats per minute, blood pressure of 120/70 mm Hg, and respiratory rate of 18 breaths per minute. Abdominal examination revealed tenderness and guarding in the left upper quadrant with a palpable, firm mass. No peritoneal signs were noted. Bowel sounds were present, and there was no evidence of hepatosplenomegaly. The remaining systemic examination was unremarkable.

Given the severity of the abdominal pain and the palpable mass, further diagnostic work-up was initiated. Initial laboratory investigations revealed a normal complete blood count, liver function tests, and renal function. However, a markedly elevated serum amylase level of 480 U/L (normal range: 30-110 U/L) and lipase level of 620 U/L (normal range: 23-300 U/L) raised suspicion of a pancreatic etiology. To explore this further, additional investigations were performed, including serum CA 19-9, which returned within the normal range, effectively ruling out pancreatic malignancies.

**Figure 1:**
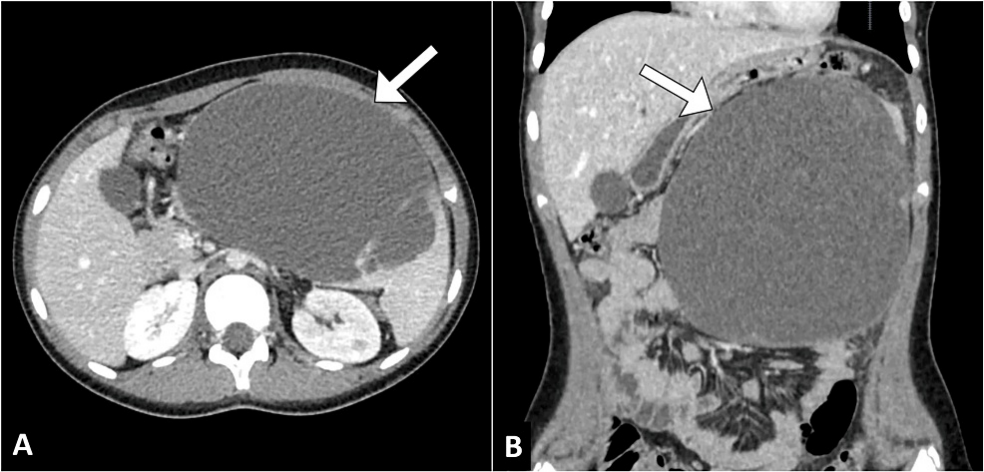
Axial (A) and coronal (B) images from a CT of the abdomen illustrate a large unilocular cystic lesion (arrow) within the spleen, prominently exhibiting mass effect.

In view of the abdominal pain and palpable mass, the patient was admitted to the pediatric surgery service for further evaluation and management. A contrast-enhanced computed tomography scan of the abdomen and pelvis was performed to investigate the underlying cause. The computed tomography scan confirmed the presence of a large cystic lesion in the spleen, without evidence of vascular invasion or lymphadenopathy (Figure 1).

**Figure 2:**
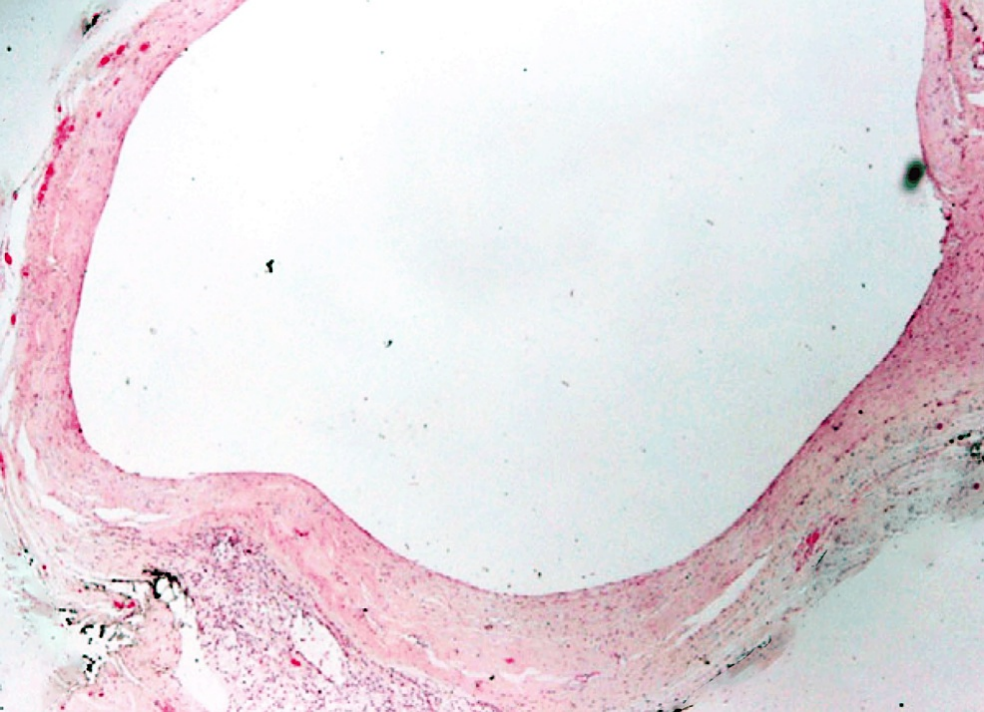
Histopathological image showing the epithelial-lined cyst.

The differential diagnosis at this point included various splenic lesions such as an epidermoid cyst, hydatid cyst, lymphangioma, or pseudocyst. The decision was made to proceed with surgical intervention due to the size of the cyst and the patient's symptoms. The patient underwent a laparoscopic splenectomy, and the resected specimen confirmed the diagnosis of a benign splenic cyst (Figure 2).

Following the surgical procedure, the patient's recovery proceeded without any noteworthy complications. She exhibited an uneventful postoperative course, which encompassed the absence of unexpected or adverse events. On the third day after the operation, she was deemed fit for discharge. As part of her post-discharge care, the patient received comprehensive guidance regarding the necessity of appropriate vaccinations and antibiotic prophylaxis. These precautions were advised due to the absence of a functioning spleen, which increases susceptibility to certain infections.

In addition to these immediate postoperative measures, a robust long-term follow-up plan was established. This plan included regular, scheduled appointments to monitor the patient's progress and well-being over an extended period. Over the course of two years of follow-up, the patient remained in good health, with no notable complications or concerns related to the splenectomy. This demonstrates the effectiveness of the initial management and the patient's ongoing well-being in the absence of her spleen.

## Discussion

Splenic cysts are rare clinical findings, with an incidence of approximately 0.07% in the general population. They are classified into primary (true) and secondary (false) cysts based on the presence or absence of a cellular epithelial lining. Primary cysts can further be divided into parasitic and non-parasitic cysts based on their etiology [2]. While splenic cysts are uncommon in the United States, they are more prevalent in regions where Echinococcus is endemic, such as the Mediterranean area, New Zealand, Australia, and South America [1].

In the pediatric population, congenital splenic cysts are the most common type, while pseudocysts are infrequent [1,2]. These cysts often remain asymptomatic and are incidentally diagnosed, with symptoms typically manifesting during childhood or adolescence as they grow or lead to complications [2]. The clinical presentation in our case, characterized by acute abdominal pain and a palpable mass, aligns with the reported symptoms of splenic cysts, highlighting the importance of early recognition and management [3].

Various pathophysiological mechanisms have been proposed to explain the development of splenic cysts in pediatric patients. The mesothelial invagination theory suggests that congenital cysts form when the mesothelial lining invades the capsule during development, allowing for metaplasia and fluid secretion within the cyst. Congenital cysts may also arise from the invasion of the peritoneum along with its mesothelial lining after splenic capsule rupture or entrapment of mesothelial cells in splenic sulci [3,4].

Another theory, the lymph space theory, suggests that cysts can originate from the spleen's normal lymphatic spaces. The endodermal inclusion theory proposes that epithelial splenic cysts develop through metaplasia of a heterotopic endodermal inclusion within the spleen, leading to cysts with various types of epithelial lining. These pathophysiological mechanisms underscore the complex and varied nature of splenic cyst development [4,5].

Clinical presentation often includes abdominal pain, as observed in our case, with approximately 46% of patients reporting discomfort typically in the left upper quadrant [3]. Other associated symptoms and signs may include a palpable mass, thrombocytopenia, early satiety, abdominal swelling, distension, nausea, vomiting, changes in bowel function, weight loss, and other nonspecific gastrointestinal complaints. Timely diagnosis and appropriate management are essential to alleviate symptoms and prevent potential complications, especially in pediatric patients where splenic preservation is a primary consideration [5].

In a retrospective study by Hodge et al. [6], the authors provide a comprehensive exploration of pediatric splenic cysts spanning an 18-year period and encompassing 21 pediatric cases. Their study reveals a significant 3:1 female-to-male ratio among pediatric patients diagnosed with splenic cysts. Furthermore, the study highlights the increasing preference for spleen-preserving surgical techniques, such as partial splenectomy, over total splenectomy, aimed at safeguarding vital immune functions while mitigating the potential for cyst recurrence.

## Conclusions

In conclusion, this case report underscores the significance of early recognition and prompt surgical intervention in the management of splenic cysts, especially in pediatric patients. Splenic cysts, although rare, can manifest with a range of symptoms, including acute abdominal pain, and their diverse etiologies and pathophysiological mechanisms necessitate a comprehensive diagnostic approach. Successful outcomes, as observed in this case, highlight the importance of timely evaluation and treatment, emphasizing that a high index of suspicion, multidisciplinary collaboration, and appropriate surgical management can lead to the resolution of symptoms and ensure the long-term well-being of the patient.
